# Evaluation of the BioFire^®^ FilmArray^®^ Blood Culture Identification Panel on positive blood cultures in a regional hospital laboratory in KwaZulu-Natal

**DOI:** 10.4102/ajlm.v5i1.411

**Published:** 2016-09-30

**Authors:** Mokshanand Fhooblall, Fikile Nkwanyana, Koleka P. Mlisana

**Affiliations:** 1 School of Laboratory Medicine and Medical Sciences, University of KwaZulu-Natal, Durban, South Africa; 2 Howard College, University of KwaZulu-Natal, Durban, South Africa; 3 Department of Medical Microbiology, National Health Laboratory Services, Durban, South Africa

## Abstract

**Background:**

There are presently many non-culture-based methods commercially available to identify organisms and antimicrobial susceptibility from blood culture bottles. Each platform has its benefits and limitations. However, there is a need for an improved system with minimal hands-on requirements and short run times.

**Objectives:**

In this study, the performance characteristics of the FilmArray^®^ BCID Panel kit were evaluated to assess the efficiency of the kit against an existing system used for identification and antimicrobial susceptibility of organisms from blood cultures.

**Methods:**

Positive blood cultures that had initially been received from hospitalised patients of a large quaternary referral hospital in Durban, South Africa were processed as per routine protocol at its Medical Microbiology Laboratory. Positive blood cultures were processed on the FilmArray BCID Panel kit in parallel with the routine sample processing. Inferences were then drawn from results obtained.

**Results:**

Organism detection by the FilmArray BCID panel was accurate at 92.6% when organisms that were on the repertoire of the kit were considered, compared to the combination methods (reference method used in the study laboratory). Detection of the antimicrobial resistance markers provided by the panel and reference method demonstrated 100% consistency. Blood cultures with a single organism were accurately identified at 93.8% by FilmArray, while blood cultures with more than one organism were identified at 85.7%.

**Conclusion:**

The FilmArray BCID Panel kit is valuable for detection of organisms and markers of antibiotic resistance for an extensive range of organisms.

## Introduction

Blood cultures are a crucial element in the diagnostic workup of newly-admitted patients and for monitoring suspected bloodstream infection in inpatients.^1^ Identification of organisms responsible for bacteraemia and/or fungaemia, as well as antimicrobial susceptibility patterns of these organisms, are currently done from blood cultures via various methods. Many systems (phenotypic and genotypic methods) exist for workup of blood cultures.^1,2^ However, shortcomings in identification of microorganism(s) from blood cultures do still exist.^1^ There is a need for a system or combination of systems that offer a compromise between accuracy, precision and timing of reporting of organisms from positive blood cultures. Timely and appropriate institution of antibiotics following the susceptibility report of isolated organisms from the laboratory should be also made possible.^1^ This would be in line with present recommendations of antibiotic stewardship programmes and control of transmission of multidrug-resistant organisms.

Ideally, for organism identification and susceptibility pattern derivation from blood cultures, a system should have a turnaround time of less than two hours and minimal hands-on time.^1^ The available genotypic and phenotypic systems, both nucleic acid- and non-PCR-based, are attractive options, although each has its specific limitations.^3,4^ Current systems do not offer a comprehensive range of organisms, with some detecting only bacteria but not detecting yeasts. Not all systems offer antimicrobial susceptibility information and not every laboratory has adequate and/or skilled personnel to prepare samples for running on molecular kits.^1^

Hands-on time with samples refers to the actual time spent in preparing the run for the samples to be tested.^1^ Run time refers to the interval between setting up the assay and obtaining a result fit for reporting.^3^ Resistance genes refer to gene sequences present in the organisms that may or may not be expressed. Expression of resistance phenotypes may be seen as lack of susceptibility to certain antimicrobials.^3^

The FilmArray^®^ BCID Panel (bioMérieux Clinical Diagnostics, Salt Lake City, Utah, United States) is a PCR-based molecular platform,^5^ and high analytical sensitivities of over 90% have been reported by other studies.^6,7,8,9,10^ It can potentially identify 19 bacteria, five yeasts and three antibiotic resistance markers from positive blood culture bottles. The FilmArray panel has been studied by Blaschke et al.,^6^ Altun et al.,^7^ Rand and Delano,^8^ Bhatti et al.,^9^ Southern et al.^10^ and Ward et al.^11^ In the different studies, FilmArray was compared to various methods for organism detection rate and time taken for organism identification, in view of improving current identification and susceptibility testing.^6,7,8,9,10,11^ It would be highly beneficial to have identification and susceptibility reports within 60 minutes of a bottle flagging positive, as proposed by the FilmArray package insert. With our present combination methods, a minimum of 24 hours would be needed from the time a blood culture becomes positive to reporting identification and resistance patterns.

This study was undertaken to assess FilmArray, as an option to improve on blood culture reporting. The plethora of bacteria and yeasts covered by the FilmArray panel is frequently recovered in blood cultures. The genetic mutations that were available as antimicrobial resistance genes are also of interest. In a quaternary reference hospital, organisms recovered were from patients exposed to a variety, and possibly suboptimal doses, of antibiotics as they are referred from various clinics across KwaZulu-Natal. This forms a perfect niche for multidrug-resistant organisms. Carbapenems, amongst others, are extensively used in private and public sectors in KwaZulu-Natal, and estimates of *Klebsiella pneumoniae-*type carbapenemase (KPC) resistance gene are needed. The KPC gene, amongst others, offered by FilmArray would be interesting to consider from that perspective.

## Methods

### Ethical considerations

Ethics approval was obtained from the Biomedical Research Ethics Committee of the University of KwaZulu-Natal (BE456/14) and the study was done on blood cultures received by the laboratory. Consent was obtained from the patients whose samples were used as per the existing agreement between the Durban National Health Laboratory Services Medical Microbiology laboratory, the University of KwaZulu-Natal and the hospital where the study was undertaken.

### Study setting and design

The study was conducted at the quaternary-level facility for the province of KwaZulu-Natal, South Africa, a regional referral hospital with a bed capacity of 846. Becton Dickinson BACTEC (Becton Dickinson, United States) blood cultures were received as per routine work from all wards and intensive care units of the hospital covered by the Medical Microbiology Laboratory of the National Health Laboratory Services, Durban, KwaZulu-Natal.

Per routine processes, the blood cultures are loaded into the automated blood culture continuous monitoring system BACTEC™ FX System (BD Diagnostics, Franklin Lakes, New Jersey, United States). They remain incubated in the instrument pending organism growth and detection. After five days of no signal received from a blood culture bottle, the bottle is retrieved and resulted as ‘no growth after five days’. Blood cultures that become positive are retrieved and worked up by Gram stain and subculture. Plates from subculture are interpreted by microbiologists and further biochemical or automated tests are utilised. Ultimately, positive blood cultures are reported with organism identity and susceptibility.

In this study, blood cultures received between February and April 2015 that became positive were evaluated using a prospective analytical approach. During the study, there was no request made to wards to send more blood cultures or to restrict the number of blood cultures made available for the study. Any blood culture that became positive was included in the study. The number of positive blood cultures from the same patient, patient name, patient clinical diagnosis or antibiotic received by patient with positive blood culture were not used as exclusion criteria.

Data on positive blood culture results were drawn from our laboratory information system (LIS) – TrakCare Lab, version 6.10, InterSystems Corporation (Cambridge, Massachusetts, United States). The BD BACTEC FX system used for blood cultures and the VITEK^®^ 2 microbial ID/AST testing system (bioMérieux Clinical Diagnostics, United States) used by our combination methods were interfaced to the LIS. Data on identification and susceptibility of organisms recovered by FilmArray were recorded into Microsoft Excel 2013 (Microsoft, Redmond, Washington, United States). Identification and susceptibility patterns of organisms recovered from positive blood cultures by combination methods were drawn from the LIS and corresponding blood cultures run on FilmArray were drawn and compared. Reproducibility was achieved by retesting five random positive blood cultures once each on FilmArray. The results from the first run on the instrument were compared to the second run. Four external controls were used as external standards to confirm the good running of the FilmArray panel for this study.

### External controls

American Type Culture Collection (ATCC) strains were used as external controls and set up by both subculture and FilmArray. Strains used (all obtained from Davies Diagnostics, Randburg, South Africa) included: *Listeria monocytogenes* ATCC 13932, *Klebsiella pneumoniae* ATCC 1705, *Candida kruzei* ATCC 6758, *Pseudomonas aeruginosa* ATCC 27853, *Candida tropicalis* ATCC 66029, *Hemophilus influenzae* type B ATCC 33533 and *Candida glabrata* ATCC MYA 2956. These ATCC strains were used for quality control in the laboratory as internal quality control for bench controls and were performed weekly. Pure colonies of these strains were inoculated into brain heart infusion broth and incubated for 30 minutes. Using an optical densitometer, a 0.5 McFarland standard suspension was made from this brain heart infusion broth and injected into sterile blood cultures. These blood cultures were loaded into the BD BACTEC FX system and retrieved when positive. One hundred microlitres of the contents of the positive blood culture were aspirated and mixed with sample buffer (provided in the kit). The pouch was loaded onto the FilmArray instrument and the result read later. The controls – in the form of ATCC strains spiked in blood cultures – were run randomly in between the runs of the positive blood cultures from patients on the FilmArray instrument. Four positive blood cultures (with a total of seven ATCC strains) were run on FilmArray, dispersed between 101 positive blood cultures that were received from hospital. This represented an average of 0.04 controls per assay run.

### Combination methods

The usual protocol of blood culture workup in the study’s laboratory constituted the *combination methods*. These were used collectively in this study as the reference method. After a blood culture bottle became positive, drops from its contents were smeared on glass slides, air-dried and Gram stained. This was followed by microscopy of the smear. Based on the morphology of the organism seen on the Gram stain, different culture media were inoculated and incubated under appropriate temperature and atmospheric conditions for at least 18 hours.

For gram-positive cocci in clusters, blood agar, mannitol-salt agar and DNA plates were set up. For gram-positive cocci in chains, blood agar with optochin disc, MacConkey agar and bile esculin agar were set up. For small gram-positive bacilli, blood agar and bile esculin agar were set up. For large gram-positive bacilli, blood agar and egg yolk agar plates were set up. For gram-negative bacilli, chocolate agar and MacConkey agar were set up. For gram-negative cocci, chocolate agar and MacConkey agar were set up. For yeasts, blood agar and Sabouraud’s dextrose agar were set up. Interpretation and further testing of recovered organism(s) were done the next day.

Colonies of organisms were tested by: catalase, indole and oxidase tests; staphylococcal latex agglutination tests; and streptococcal grouping assays. Germ tube tests were done on suspected yeasts colonies. Suspensions were made of the colonies and set up for API 20E, API 20NE, API 20 Strep, API NH, API Coryne, API 20 C AUX (bioMérieux Clinical Diagnostics, United States) and the VITEK 2 microbial ID/AST testing system (bioMérieux Clinical Diagnostics, United States). Antibiotic susceptibility testing was done using the Kirby-Bauer disk-diffusion testing system for oxacillin resistance screening, screening of extended-spectrum beta-lactamases (ESBLs) and Modified-Hodge test screening. ESBLs are enzymes that confer resistance to many beta-lactam antibiotics.^12^ Antimicrobial gradient test (Etest) was also used for minimum inhibitory concentration evaluation. Minimum inhibitory concentration is the lowest concentration of antibiotic that inhibits discernable growth of an organism following overnight incubation. It permits broad differentiation into categories of susceptibility and nonsusceptibility.^5^ The identification and susceptibility reports were read the next day. The date and time each blood culture became positive and the actual date and time report of blood culture was reported were recorded in Microsoft Excel for all blood cultures included in the study. The different times taken for reporting positive blood cultures were compared and the minimum turnaround time determined for positive cultures by combination methods.

### FilmArray BCID panel

Each positive blood culture was run on a single FilmArray kit within eight hours of becoming positive. The positive blood culture was Gram-stained, subcultured and simultaneously run on FilmArray. One hundred microlitres of the contents of the blood culture bottle were aspirated, mixed with sample buffer (provided) and loaded into the FilmArray pouch. The pouch was loaded onto the FilmArray instrument, which was connected to a computer system. Using the FilmArray software interface, the different steps of DNA extraction, nested and multiplex PCRs and post-amplification analysis may be visualised and timed. At the end of the run, a report was automatically generated which documented it as having any detectable organism(s) as well as any antimicrobial resistance gene(s) – *mecA, vanA/B* and KPC. Once a positive blood culture was setup on FilmArray, a timer was started and time until report generation was noted and compared to the time recorded in the printout report from the FilmArray instrument. Validity of results was also included in the report, with invalid results reported if either of the two in-built internal controls failed.

### Statistical analyses

In this study, identification and antimicrobial susceptibility of organisms obtained by the FilmArray panel were compared to those obtained by combination methods. Identification that was brought down to the nearest precision possible (genus/species/complex/subspecies level achievable) by FilmArray with respect to the results seen with combination methods was labelled as ‘precisely identified’; organisms that were missed or misidentified (genus/species/complex/subspecies level), despite being on the panel, were labelled as either ‘missed’ or ‘misidentified’. The number of organisms, rather than the number of blood cultures, was used to evaluate the performance of FilmArray in the statistical analyses.

Sensitivity, specificity, positive predictive value and negative predictive value as well as agreement were analysed. Calculations of sensitivities, specificities, positive predictive values and negative predictive values were adjusted according to results obtained. ‘True positive’ was defined as an organism on the FilmArray repertoire, identified by combination methods and also accurately identified by FilmArray. ‘True negative’ was defined as any microorganism not on the FilmArray repertoire and not detected by FilmArray. ‘False positive’ describes any microorganism identified by the FilmArray that was not mentioned in the FilmArray kit specifications because of design limitations. ‘False negative’ was any microorganism on the FilmArray repertoire that was identified by combination methods but was reported by FilmArray as any result other than the correct identification (missed/misidentified). Organisms detected by FilmArray while not identified by combination methods were excluded from the number of organisms detected by FilmArray in our calculations. This was due to the assumption that the combination methods was the gold standard method. The combination methods made use of trusted and proven phenotypic methods; and would be (in theory) superior to the FilmArray panel for organism detection. Calculations were done using IBM SPSS Statistics for Windows (Version 22.0; IBM Corp, Armonk, New York, United States).

## Results

Over the study period, 2119 blood cultures were received by the laboratory and 22.3% (472/2119) were positive. A total of 113 positive blood cultures were worked up both by combination methods and FilmArray kits.

Three blood culture bottles were reported as invalid by the FilmArray instrument. Four blood culture bottles were used as external controls on FilmArray by inoculation with known ATCC organisms. All four external controls yielded the desired identification. Five blood culture bottles were randomly repeated on FilmArray to evaluate reproducibility, and all five gave the same results each time they were run.

This permitted the actual evaluation of 101 positive blood culture bottles containing clinical isolates, comparing FilmArray to combination methods. Overall, 101 positive blood cultures were tested; 92.1% (93/101) with one organism and 7.9% (8/101) with more than one organism by the combination methods. All positive blood cultures included in the study had organisms detectable on initial Gram stain. In addition, all 101 positive bottles were identified down to the presence of one or more organisms by combination methods. In total, 109 organisms were detected by combination methods from 101 positive cultures. This is because some blood cultures had one organism, and other blood cultures had more than one organism detected. Of the organisms, 86.2% (94/109) were on the repertoire of the FilmArray panel for potential detection.

**FIGURE 1:**
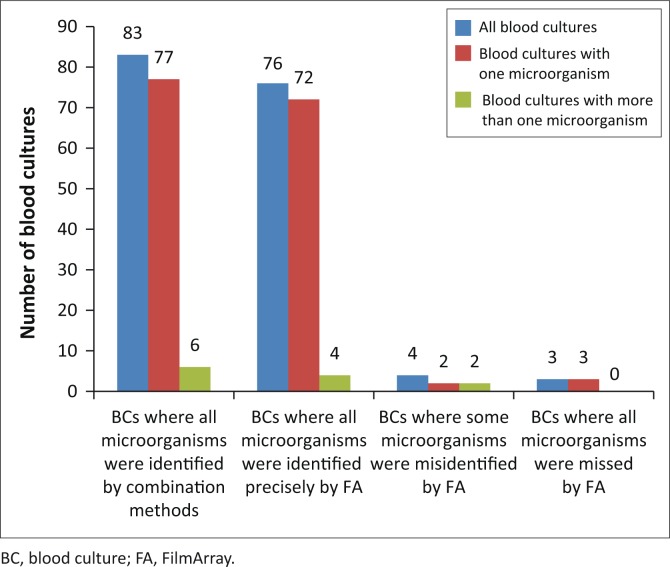
Comparison of blood culture results using combination methods against FilmArray.

The performance of FilmArray on positive blood cultures is described (blood culture-wise) in Figure 1. With 91.6% (76/83) organisms correctly identified as part of the FilmArray repertoire we found equally acceptable accuracy of the FilmArray BCID panel, organism wise: 92.6% (87/94) organisms identified that were identifiable (as per the repertoire claimed by the manufacturer) compared to combination methods. Performance of FilmArray on blood cultures with one type of organism and blood cultures with more than one organism is further elaborated in Table 1. Organisms and resistance genes recovered by FilmArray are in Tables 2 and 3 – where, notably, the majority of organisms recovered were Gram-positive organisms. Conflicting results between FilmArray and combination methods are shown in Table 4. FilmArray missed and/or misidentified 7.5% (7/94) of the organisms.

**TABLE 1:** Performance characteristics of FilmArray compared to combination methods.

Parameter	All positive blood cultures (*n* = 101)	Positive cultures with one type of organism (93/101)	Positive cultures with more than one type of organism (8/101)
Sensitivity	92.6%, 95% CI [86.1–96.7]	93.8%, 95% CI [87–97.7]	85.7%, 95% CI [62.1–97.5)
Specificity	100%, 95% CI [n/a]	100%, 95% CI [n/a]	100%, 95% CI [n/a]
Positive predictive value	100%, 95% CI [n/a]	100%, 95% CI [n/a]	100%, 95% CI [n/a]
Negative predictive value	68.2%, 95% CI [47.5–84.9]	72.2%, 95% CI [49.5–89]	50%, 95% CI [10.7–89.3]
Cohen’s Kappa	0.774	0.807	0.6

Positive blood cultures described are organisms that were on the FilmArray BCID repertoire.

**TABLE 2:** Performance of FilmArray on blood cultures with one type of organism – by microorganism and antibiotic resistance marker

Identification by combination methods	Number detected	FilmArray		

		Precisely identified *n* (%)	Misidentified *n* (%)	Missed *n* (%)
**Gram-positive bacteria**
Methicillin-resistant *Staphylococcus aureus*	4	4 (100.0)	-	-
Methicillin-resistant *Staphylococcus* species	37	37 (100.0)	-	-
Methicillin-sensitive *Staphylococcus aureus*	7	7 (100.0)	-	-
Methicillin-sensitive *Staphylococcus* species	3	1 (33.3)	2 (66.7)	-
*Streptococcus* spp.	2	1 (50.0)	-	1 (50.0)
*Streptococcus agalactiae*	0	-	-	-
*Streptococcus pneumoniae*	1	1 (100.0)	-	-
*Streptococcus pyogenes*	0	-	-	-
*Enterococcus* spp.	4	3 (75.0)	-	1 (25.0)
*Listeria monocytogenes*	0	-	-	-
**Total Gram-positive bacteria on panel**	**58**	**54 (93.1)**	**2 (3.4)**	**2 (3.4)**
**Gram-negative bacteria**
*Acinetobacter baumannii*	3	2 (66.7)	-	1 (33.3)
*Enterobacter cloacae* complex	1	1 (100.0)	-	-
*Escherichia coli*	3	3 (100.0)	-	-
*Klebsiella pneumoniae*	8	8 (100.0)	-	-
*Klebsiella oxytoca*	0	0	-	-
*Proteus* spp.	0	0	-	-
*Pseudomonas aeruginosa*	3	3 (100.0)	-	-
*Serratia marcescens*	2	2 (100.0)	-	-
*Haemophilus influenzae*	0	0	-	-
*Neisseria meningitidis*	0	0	-	-
*Enterobacteriaceae* spp.	0	0	-	-
**Total Gram-negative bacteria on panel**	**20**	**19 (95.0)**	**-**	**1 (5.0)**
**Yeasts**				
*Candida albicans*	2	2 (100.0)	-	-
*Candida glabrata*	0	0	-	-
*Candida krusei*	0	0	-	-
*Candida parapsilosis*	0	0	-	-
*Candida tropicalis*	0	0	-	-
Total yeasts on panel	2	2 (100.0)	-	-
**Total identifiable bacteria and yeasts**	**80**	**75 (93.8)**	**4 (5.0)**	**3 (3.8)**
**Antibiotic resistance genes**
*mecA*	41	41	-	-
*vanA/vanB*	1	1	-	-
KPC	0	0	-	-

KPC, *Klebsiella pneumoniae* carbapenemase.

**TABLE 3:** Performance of FilmArray on blood cultures with more than one type of organism – by microorganism and antibiotic resistance marker.

Identification by combination methods	Number detected	FilmArray		

		Precisely identified	Misidentified	Missed
**Gram-positive bacteria**
Methicillin-resistant *Staphylococcus aureus*	0	-	-	-
Methicillin-resistant *Staphylococcus* species	3	3 (100.0)	-	-
Methicillin-sensitive *Staphylococcus aureus*	1	1 (100.0)	-	-
Methicillin-sensitive *Staphylococcus* species	0	-	-	-
*Streptococcus* spp.	3	2 (66.7)	1 (33.3)	-
*Streptococcus agalactiae*	1	1 (100.0)	-	-
*Streptococcus pneumoniae*	0	-	-	-
*Streptococcus pyogenes*	0	-	-	-
*Enterococcus* spp.	3	2 (66.7)	-	1 (33.3)
*Listeria monocytogenes*	0	-	-	-
**Total Gram-positive bacteria on panel**	**11**	**9 (81.8)**	**1 (9.1)**	**1 (9.1)**
**Gram-negative bacteria**
*Acinetobacter baumannii*	0	-	-	-
*Enterobacter cloacae* complex	0	-	-	-
*Escherichia coli*	1	1 (100.0)	-	-
*Klebsiella pneumoniae*	0	-	-	-
*Klebsiella oxytoca*	0	-	-	-
*Proteus* spp.	1	1 (100.0)	-	-
*Pseudomonas aeruginosa*	1	1 (100.0)	-	-
*Serratia marcescens*	0	-	-	-
*Haemophilus influenzae*	0	-	-	-
*Neisseria meningitidis*	0	-	-	-
*Enterobacteriaceae* spp.	0	-	-	-
**Total Gram-negative bacteria on panel**	**3**	**3 (100%)**	**-**	**-**
**Yeasts**
*Candida albicans*	0	-	-	-
*Candida glabrata*	0	-	-	-
*Candida krusei*	0	-	-	-
*Candida parapsilosis*	0	-	-	-
*Candida tropicalis*	0	-	-	-
Total yeasts on panel	0	-	-	-
**Total identifiable bacteria and yeasts**	**14**	**12 (85.7)**	**1 (9.1)**	**1 (9.1)**
**Antibiotic resistance genes**
*mecA*	3	3	-	-
*vanA/vanB*	0	0	-	-
KPC	0	0	-	-

KPC, *Klebsiella pneumoniae* carbapenemase.

**TABLE 4:** Breakdown of conflicting results.

Sample	Blood culture	Combination methods	FilmArray
1	One type of organism	*Enterococcus* spp.	Missed
2	One type of organism	*Streptococcus* spp.	Missed
3	One type of organism	Methicillin-sensitive *Staphylococcus* spp.	Methicillin-sensitive *Staphylococcus aureus* (Misidentified)
4	One type of organism	Methicillin-sensitive *Staphylococcus* spp.	Methicillin-sensitive *Staphylococcus aureus* (Misidentified)
5	One type of organism	*Acinetobacter baumanii*	Missed
1	More than one type of organism	*Enterococcus* spp.†	Missed
2	More than one type of organism	*Streptococcus* spp.‡	*Enterococcus* spp. (Misidentified)

† , Sample was found to have methicillin-resistant *Staphylococcus* spp. on subculture as detected by the FilmArray;

‡ , Sample was found to have methicillin-resistant *Staphylococcus* spp. on subculture as detected by the FilmArray; along with misidentification of *Streptococcus* spp. as *Enterococcus* spp.

A minimum of 24 hours was needed by combination methods to obtain results from positive blood cultures. A maximum of 65 minutes was needed when using FilmArray for obtaining identification and potential susceptibility information of an organism from a positive blood culture. Once the kit was manually set up and loaded on the FilmArray instrument, the run, taking 55–60 minutes preceding report generation, was all automated, needing no operator intervention.

Organisms recovered that had resistance genes undetectable by FilmArray included: ESBL-producing *Klebsiella pneumoniae*, ESBL-producing *Enterobacter cloacae*, ESBL-producing *Escherichia coli*, ESBL-producing *Proteus mirabilis* and carbapenemase-producing *Acinetobacter baumannii* (also producing metallobetalactamases and oxacillinases).

In certain blood cultures run, there was another form of discrepancy – where more organisms were detected by FilmArray than the combination methods. These included 14 isolates comprising these organisms: *Enterobacter cloacae* complex, *Proteus* spp., *Serratia marcescens, Acinetobacter baumannii, Haemophilus influenzae, Escherichia coli, Enterococcus* spp., *Streptococcus* spp., *Candida parapsilosis* and *Staphylococcus aureus*.

## Discussion

Good correlation was seen between the FilmArray and combination methods for identification of organisms and resistance genes. An overall accurate identification rate of 92.6% was achieved for organisms identifiable by FilmArray from all blood cultures. This was slightly superior to the 91% sensitivity achieved overall in a separate study by Blaschke et al., which used a developmental version of the FilmArray,^6^ but slightly below sensitivities found by other studies.^7,8,9,10,11^

Higher sensitivity of FilmArray was seen with positive cultures with one type of organism (93.8% [75/80] organisms detected) than with cultures with more than one organism (85.7% [12/14] detected) in our study. This corroborated with a higher proportion of organisms not accurately identified (either missed or misidentified) from cultures with more than one organism tested by FilmArray. This was in line with previous work done on the FilmArray BCID, which also found lower sensitivity for cultures that detected more than one organism.^6,7,8,10^ Nonetheless, while detection of organisms from blood cultures with one type of organism appears better overall, organisms that were missed by FilmArray in our study were more from blood cultures with one type of organism. Three out of four false negatives reported were seen from blood cultures with one type of organism. That was in correlation with Blaschke et al., who also had a higher number of false negatives from blood cultures with one type of organism as compared to blood cultures with more than one organism (37.5%).^6^

Of concern were organisms that were on the repertoire of the FilmArray but not detected and still picked up by combination methods. If FilmArray were applied, these results would have been erroneously interpreted as negative. Overall, over 7% of organisms (7/94) were falsely classified as negative in our study, although it did include *Enterococcus* spp., which was also missed in two blood cultures. This false-negative rate was higher than findings from other studies which reported lower rates.^6,8,10,11^

*Enterococcus* spp. was not detected by FilmArray from two blood cultures in our study. This is significant, as some *Enterococcus* spp. bloodstream infections carry a high mortality rate, necessitating early and appropriate antibiotic infusion, especially in those with suspected infective endocarditis. *Enterococcus* spp. was also amongst those missed by FilmArray in both Blaschke et al. (*n* = 1)^6^ and Altun et al. (*n* = 2).^7^ Misidentification of methicillin-sensitive *Staphylococcus* spp. as methicillin-sensitive *Staphylococcus aureus* was observed on two blood cultures (blood cultures with one type of organism) in our analysis. This was a species classification mistake by FilmArray and delivery of such results might lead to overtreatment with cloxacillin. Similar mislabelling has been observed in other studies.^9,10^ There was negative misclassification of *Staphylococcus* (at genus level) by FilmArray in our study, which was in contrast to findings by Blaschke et al. and Altun et al., where *Staphylococcus* was missed by FilmArray while recovered by culture.^6,7^

*Streptococcus* spp. was erroneously identified as *Enterococcus* spp. by FilmArray on two instances in blood cultures in our analysis. This type of misclassification could lead to the wrong choice of antibiotic, as therapy choices for these two are dichotomous.

During our investigation, *Candida albicans* was the only yeast recovered from patient blood culture in two instances and was also accurately identified by the FilmArray. Congruous findings of 100% identification rate of yeasts were also seen by Altun et al.^7^ and Southern et al.^10^ This was as opposed to findings by Blaschke et al. where only 75% sensitivity was achieved for *Candida albicans.*^6^

Antibiotic resistance marker detection by FilmArray was not an issue in our study. All *mecA* and *vanA/B* genes detected corresponded with culture and Vitek results for the respective samples. The KPC gene was not detectable in the clinical samples tested and hence could not be commented on. *mecA* gene detection was inaccurate in previous studies, with Bhatti et al. detecting it by FilmArray, when not actually present, in four specimens.^9^ Ward et al. also reported six spurious instances of *mecA* detection from both methicillin-sensitive *Staphylococcus* spp. and methicillin-sensitive *Staphylococcus aureus*.^11^ However, lack of detection of *mecA* was also observed from nine coagulase-negative methicillin-resistant *Staphylococcus* species in their study.

Minimal laboratory personnel training would be needed for incorporation of FilmArray into the routine daily workflow of the laboratory. The stand-alone component of FilmArray during its 55–60 minute run time is beneficial. If implemented, this would permit attention to other laboratory work pending the report on positive blood culture. The FilmArray instrument software interface also enabled quick troubleshooting and can be coupled to a laboratory information system for more efficient updates of results. In addition, when the FilmArray pouch is poorly rehydrated, the system immediately informs laboratory personnel so that an entire hour is not wasted. Finally, the 65 minutes required for running FilmArray on a positive blood culture was substantially shorter than the time required to run the combination methods (a minimum of 24 hours).

### Limitations

FilmArray covered only three types of genetic markers (*mecA, vanA/B* and KPC). Other mechanisms of resistance detected by combination methods, such as ESBLs, were not provided by FilmArray and were therefore missed. As such, an efficient comprehensive antibiotic susceptibility system may be needed with this system.

In certain blood cultures, detection of organisms by FilmArray was in excess of that of combination methods. These discrepant results were not included in calculations but may have been due to poor performance of combination methods or detection of nucleic acid from non-viable organisms by FilmArray in the blood culture.^1^

Our sample size with regard to assessing performance characteristics of FilmArray was small at 101. Increasing the pool of positive blood cultures tested, with a more diverse range of isolates, would have been beneficial.

### Recommendations

One positive blood culture can be run at a time on the FilmArray instrument. Based on the aforementioned rapid reporting times of this instrument, FilmArray would be appropriate to reduce turnaround time in a microbiology laboratory of a hospital. The unavoidable delays in testing more than one positive blood culture could be overcome by using at least two units of the FilmArray instrument. The identity of the organism, along with clinical information, would help clinical microbiologists and clinicians rule out whether a positive blood culture contains a contaminant or an actual pathogen, necessitating start or change of therapy.^2,4^

### Conclusion

Our assessment of the FilmArray panel on positive blood cultures demonstrated reasonable accuracy and practical benefits. Our results were in agreement with the reference method used in this study in the majority of positive blood cultures tested. Yet, for the time being, it should be used coupled to an existing identification and antibiotic susceptibility determination system. This, if utilised for rapid communication of results to the physician in the ward, would ameliorate the choice of initial antimicrobial to patients and also drastically affect patient management.
